# Visceral adipose of obese mice inhibits endothelial inwardly rectifying K^+^ channels in a CD36-dependent fashion

**DOI:** 10.1152/ajpcell.00073.2024

**Published:** 2024-04-08

**Authors:** Sabita Rokka, Masoumeh Sadeghinejad, Emma C. Hudgins, Erica J. Johnson, Thanh Nguyen, Ibra S. Fancher

**Affiliations:** Department of Kinesiology and Applied Physiology, College of Health SciencesUniversity of Delaware, Newark, Delaware, United States

**Keywords:** adipose tissue, CD36, endothelial dysfunction, Kir2.1 channels, obesity

## Abstract

Obesity imposes deficits on adipose tissue and vascular endothelium, yet the role that distinct adipose depots play in mediating endothelial dysfunction in local arteries remains unresolved. We recently showed that obesity impairs endothelial Kir2.1 channels, mediators of nitric oxide production, in arteries of visceral adipose tissue (VAT), while Kir2.1 function in subcutaneous adipose tissue (SAT) endothelium remains intact. Therefore, we determined if VAT versus SAT from lean or diet-induced obese mice affected Kir2.1 channel function in vitro. We found that VAT from obese mice reduces Kir2.1 function without altering channel expression whereas AT from lean mice and SAT from obese mice had no effect on Kir2.1 function as compared to untreated control cells. As Kir2.1 is well known to be inhibited by fatty acid derivatives and obesity is strongly associated with elevated circulating fatty acids, we next tested the role of the fatty acid translocase CD36 in mediating VAT-induced Kir2.1 dysfunction. We found that the downregulation of CD36 restored Kir2.1 currents in endothelial cells exposed to VAT from obese mice. In addition, endothelial cells exposed to VAT from obese mice exhibited a significant increase in CD36-mediated fatty acid uptake. The importance of CD36 in obesity-induced endothelial dysfunction of VAT arteries was further supported in ex vivo pressure myography studies where CD36 ablation rescued the endothelium-dependent response to flow via restoring Kir2.1 and endothelial nitric oxide synthase function. These findings provide new insight into the role of VAT in mediating obesity-induced endothelial dysfunction and suggest a novel role for CD36 as a mediator of endothelial Kir2.1 impairment.

**NEW & NOTEWORTHY** Our findings suggest a role for visceral adipose tissue (VAT) in the dysfunction of endothelial Kir2.1 in obesity. We further reveal a role for CD36 as a major contributor to VAT-mediated Kir2.1 and endothelial dysfunction, suggesting that CD36 offers a potential target for preventing the early development of obesity-associated cardiovascular disease.

## Graphical abstract

**Figure:**
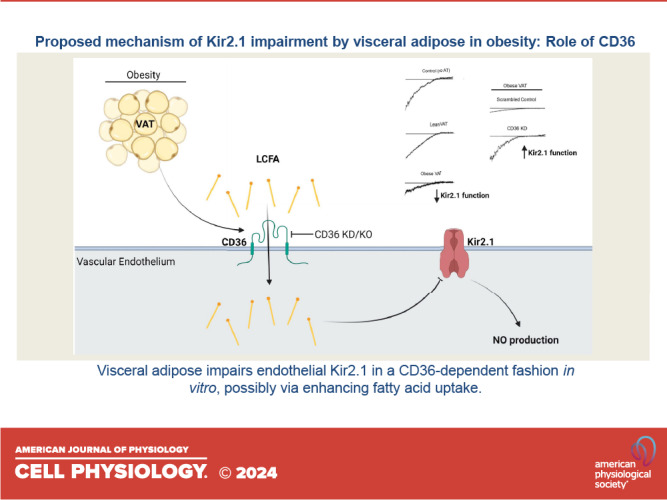

## INTRODUCTION

Adipose tissue is now recognized as a dynamic endocrine and paracrine tissue, and obesogenic conditions have been shown to differentially affect abdominal visceral (VAT) versus subcutaneous (SAT) adipose tissue (1). VAT has been shown to be particularly sensitive to obesity with increased immune cell infiltration and a concomitant increase in inflammatory markers (2–4). Recent evidence has also revealed a dichotomy in the endothelial function of adipose arteries in rodent models of obesity and in obese humans. Specifically, mesenteric and omental visceral adipose arteries exhibit robust endothelial dysfunction whereas subcutaneous adipose arteries appear unaffected, supporting the notion that altered abdominal VAT may be negatively influencing endothelial function of the embedded arteries in obesity (5–8). Obesity-induced endothelial dysfunction is a hallmark precursor to advanced cardiovascular disease driven by decreased nitric oxide (NO) production (9, 10), yet the role of VAT in mediating this outcome remains unclear.

We recently showed that inwardly rectifying K^+^ 2.1 channels (Kir2.1), upstream mediators of NO production, are functionally impaired in obesity (11, 12). We further identified that endothelial Kir2.1 channels are also robustly impaired in mesenteric visceral adipose arteries of obese mice and humans yet retain functionality in subcutaneous adipose artery endothelium, thereby indicating differential effects of obesity on endothelial Kir2.1 when located in VAT versus SAT (5). Numerous studies have established that Kir2.1 is sensitive to the cellular lipid milieu and is inhibited by lipids both directly (e.g., cholesterol and fatty acid derivatives such as long-chain fatty acid esters) and through indirect cell signaling (e.g., ceramide activation of mediators that inhibit Kir2.1) (13–16). As obesity is strongly associated with increased circulating free fatty acids (17), we, therefore, hypothesized that VAT from obese mice induces a local upregulation of endothelial CD36, the major endothelial fatty acid translocase, that results in the inhibition of Kir2.1.

CD36 is well established in promoting the formation of atherosclerotic lesions (18); however, its contributions to the earlier stages of disease processes that predate lesion formation (i.e., endothelial dysfunction) and whether CD36 expression or function is influenced by specific adipose tissues in obesity remain unknown. In contrast, elevated fatty acids are strongly linked to the onset of endothelial dysfunction in obesity and have been shown to impair NO production, induce oxidative stress, and promote inflammation (19). Therefore, in this study, we use a well-established mouse model of diet-induced obesity to determine the role of VAT in mediating endothelial Kir2.1 dysfunction via CD36 by exposing endothelial cells to VAT and SAT from lean and obese mice in vitro. We also determined if CD36 ablation restored endothelial function in a Kir2.1- and endothelial nitric oxide synthase (eNOS)-dependent fashion in VAT arteries of obese mice ex vivo using pressure myography. Our findings offer a novel insight into the role of distinct parenchymal adipose in mediating obesity-induced endothelial dysfunction.

## MATERIALS AND METHODS

### Animals

Animal studies were approved by the Institutional Animal Care and Use Committee at the University of Delaware. Ten-week-old C57BL/6J wild-type (WT) or CD36 knockout (KO) mice [The Jackson Laboratory; WT stock no. 000664; CD36 KO stock no. 019006 (B6.129S1-Cd36^tm1Mfe^/J)] were randomly divided into diet groups: lean controls maintained on a normal laboratory diet and an obese group fed a high-fat Western diet (42% kcal from fat). Respective diets were maintained for 8 wk, a time point with which endothelial dysfunction is observed in WT mice (5, 11). Mice were euthanized by cervical dislocation followed by thoracotomy. Both male and female mice were used, and data were combined where indicated. SAT from the abdominal and hindlimb regions and mesenteric VAT were isolated for use in the in vitro studies. First-order mesenteric and subcutaneous AT arteries were isolated to assess flow-induced vasodilation via pressure myography.

### Cell Culture

Human adipose microvascular endothelial cells (HAMECs) were maintained under standard culture conditions using endothelium cell medium and supplements (ScienCell). Low passage cells between P2 and P4 were used for all experiments. Cells were seeded at ∼80% confluency and allowed to become a monolayer before en bloc treatment with AT (5 mg AT/ml of culture medium) from lean or obese mice. Cells were incubated with AT for 48 h under standard culture conditions before downstream analyses. Untreated cells were used as control. To determine the effects of CD36 downregulation in vitro, HAMECs at 50% confluency were transfected with siRNA to CD36 or scrambled siRNA control using lipofectamine (RNAiMax, Invitrogen) following the manufacturer’s instructions before AT treatments.

### Patch-Clamp Electrophysiology

Following AT treatments, AT was discarded, and cells were washed two times with room temperature (RT) PBS. HAMECs were then nonenzymatically dissociated using Versene (Gibco), resuspended in bath solution, and stored on ice. Whole cell voltage clamp was performed on HAMECs using thick-walled borosilicate glass pipettes (Sutter Instruments) with resistances of 3–5 MΩ after fire polishing. Currents were recorded in a high 60 mM K^+^ bath to detect inwardly rectifying K^+^ currents using an EPC10 amplifier (HEKA Electronik) and accompanying acquisition and analysis software (Pulse and PulseFit). HAMECs were held at −30 mV and a voltage ramp of −120 to +40 mV applied over 400 ms. Currents were low‐pass filtered at 2 kHz, and recordings were digitized at 10 kHz. Before collecting data for analysis, inwardly rectifying K^+^ currents were allowed to stabilize. For recordings to be accepted for offline analysis, the patch needed to maintain a stable membrane resistance and a series resistance ≤10 MΩ for the duration of the voltage ramp protocol, which consisted of at least 20 stable recordings in a static bath. Currents were normalized to cell capacitance to obtain current densities (pA/pF) for analysis. When necessary, leak subtraction was performed offline to collect the most accurate data points at −100 mV for the group analyses.

### Western Immunoblotting

Following AT treatments, AT was discarded and HAMECs were washed three times with ice-cold PBS. Cells were then lysed in ice-cold radioimmunoprecipitation assay buffer containing protease inhibitors. Lysed samples were centrifuged to remove membrane lipids, and supernatants were stored at −80°C until use. BCA protein assays were performed to ensure equal loading of protein. Samples were reduced and denatured by boiling at 70°C for 10 min with SDS sample buffer. Samples were run on 10% SDS gels and transferred onto nitrocellulose membranes. Membranes were blocked with 5% BSA in TBS with Tween before overnight incubation with primary antibodies to Kir2.1 (1:1,000; rabbit monoclonal; Abcam, AB109750), CD36 (1:1,000; rabbit polyclonal; ThermoFisher Scientific, PA1-16813), or the loading control β‐actin (1:5,000; rabbit polyclonal; Novus Biologicals, NB600-503) at 4°C. Membranes were washed three times in TBS with Tween for a total of 30 min. Following incubations with appropriate secondary antibodies (R&D Systems Biotechne, HAF008) for 2 h at RT, membranes were washed and then incubated with enhanced chemiluminescence and imaged using the iBright FL1500 imaging system (ThermoFisher Scientific). Densitometry was performed in ImageJ (20) and Kir2.1 and CD36 densitometric values were normalized to β actin.

### Fatty Acid Uptake

A QBT fatty acid uptake kit (Molecular Devices) was used to determine fatty acid uptake in vitro. The kit uses a BODIPY-fluorescent dodecanoic acid analog previously validated to be a suitable substrate for fatty acid transporters similar to naturally occurring fatty acids and a proprietary quenching agent (21). Following AT treatments, AT was discarded and HAMECs were washed two times with RT PBS. Cells were serum starved for 1 h before incubation with fluorescent fatty acid for 90 min at 37°C. The cells were then washed and provided fresh PBS for imaging using an EVOS fluorescent microscope. Cells were imaged at ×20 magnification and at least three fields of view/per sample were collected for analysis. Following background subtraction, the mean fluorescent intensity of the entire field of view was measured in ImageJ (20), normalized to cell number in the field of view, and an average normalized mean fluorescent intensity was calculated for each sample.

### Pressure Myography

VAT and SAT arteries were isolated from respective AT depots from lean or obese WT or CD36 mice in RT HEPES buffer. Arteries were cannulated from either side onto glass pipettes in a chamber specialized for use in video microscopy, as described previously (5, 22). The chamber was placed on an Olympus CKX41 microscope equipped with a camera for visualizing the artery on a monitor, and artery diameters were measured using a model VIA-100 video system (Boekeler). The chamber was continuously perfused with oxygenated Krebs buffer maintained at 37°C. The arterial lumen was gravity perfused from both sides with Krebs buffer from graduated cylinder reservoirs that were maintained at an equal height of 60 cmH_2_O (∼44 mmHg). Arteries were maintained at physiological pressure for 1 h before preconstriction with endothelin-1 (ET-1). ET-1 was used to constrict the artery between 40% and 50% of the baseline-pressurized diameter (Table 1). Any arteries that did not constrict in this range to a maximum dose of 200 pmol/L of ET-1 were discarded from the study. Following preconstriction in the accepted range, the reservoirs were moved in equal and opposite directions to generate intraluminal flow through the vessel in a step-wise fashion. Each step increase in flow was administered for 5 min before measurement of the lumen diameter. To confirm that vascular smooth muscle function remained intact throughout the duration of the experiment, we assessed dilations to papaverine (100 µM) at the end of each protocol. If the diameter was not ≥80% of the baseline-pressurized diameter after the addition of papaverine, no measurements collected from the artery were accepted for analysis (Table 1). BaCl_2_ (30 µM) and/or *N*^ω^-nitro-l-arginine methyl ester (l-NAME; 100 μM) was added to the circulating bath after measuring the initial response to flow for 30 min before repeating the flow protocol. Dilations to flow in each artery were calculated as a percent change in dilation (%dilation) from ET-1 relative to the baseline diameter (*Eq. 1*):

(*1*) $$\%\text{Dilation }=\frac{\text{baseline diameter}-\mathrm{diameter}\;\mathrm{in}\;\mathrm{response}\;\mathrm{to}\;\mathrm{flow}\;\Delta X}{\text{baseline diameter }-\text{ diameter after preconstriction to ET-1 }}\times 100$$

**Table 1.** Vessel inclusion criteria

Artery	Average Constriction to ET-1 ± SE, %	Average Dilation to Papaverine ± SE, %
SAT-WT lean	46.6 ± 3.2	98.7 ± 0.8
VAT-WT lean	47.1 ± 4.8	98.8 ± 0.7
SAT-WT obese	48.7 ± 2.1	98.6 ± 0.7
VAT-WT obese	49.0 ± 4.2	98.7 ± 0.8
SAT-CD36 KO lean	44.9 ± 2.5	95.5 ± 4.0
VAT-CD36 KO lean	41.6 ± 3.0	98.0 ± 0.9
SAT-CD36 KO obese	44.3 ± 2.7	97.9 ± 1.4
VAT-CD36 KO obese	46.5 ± 2.3	97.7 ± 1.5

ET-1, endothelin-1; SAT, subcutaneous adipose tissue; VAT, visceral adipose tissue; KO, knockout; WT, wild type.

To generate representative traces in a subset of arteries, dilations to flow were continuously monitored through the experiment with a data point being collected every 30 s, as described previously (12, 23).

### Solutions

The bath solution for whole cell patch-clamp experiments contained the following (in mM): 80 NaCl, 60 KCl, 10 HEPES, 1 MgCl_2_, 2 CaCl_2_, and 10 glucose, pH 7.4. The pipette solution contained the following (in mM): 5 NaCl, 135 KCl, 5 EGTA, 1 MgCl_2_, 5 glucose, and 10 HEPES, pH 7.2. HEPES buffer contained the following (in mM): 145 NaCl, 5 KCl, 2 CaCl_2_, 1 MgCl_2_, 10 glucose, and 10 HEPES, pH 7.4. Krebs buffer contained the following (in mM): 123 NaCl, 4.7 KCl, 1.2 MgSO_4_, 2.5 CaCl_2_, 16 NaHCO_3_, 0.026 EDTA, 11 glucose, and 1.2 KH_2_PO_4_, pH 7.4.

### Statistical Analyses

A two-way ANOVA was used followed by Bonferroni post hoc tests, where appropriate. Two-way repeated measures ANOVA was used when comparing paired treatments across multiple groups. Nonparametric data sets were tested for significant differences using the Mann-Whitney *U* test when comparing two groups. The Kruskal-Wallis test was followed by Dunn’s post hoc test for comparing multiple groups. Initial tests were set to *P* < 0.05 before post hoc corrections. As both male and female mice were used in our studies, we first tested for significant sex differences before combining data.

## RESULTS

### VAT from Obese Mice Reduces Endothelial Kir2.1 Function

Obesity differentially alters distinct adipose depots with abdominal VAT exhibiting a marked increase in inflammation as compared to SAT (2–4). This dichotomy parallels observations in endothelial function where local mesenteric arteries embedded in VAT are robustly impaired whereas subcutaneous adipose arteries embedded in SAT retain endothelial function (5–8). Based on our previous findings detailing that endothelial Kir2.1 channels *1*) are critical regulators of endothelial control of vasodilation via NO production (12), and *2*) also exhibit a similar profile of dysfunction in obesity [i.e., Kir2.1 is impaired in endothelium of VAT of obese mice and humans but not that of SAT or lean controls (5)], we first aimed to determine if VAT may be a mediator of endothelial Kir2.1 impairment in vitro that may underlie obesity-induced endothelial dysfunction.

**Figure 1.**
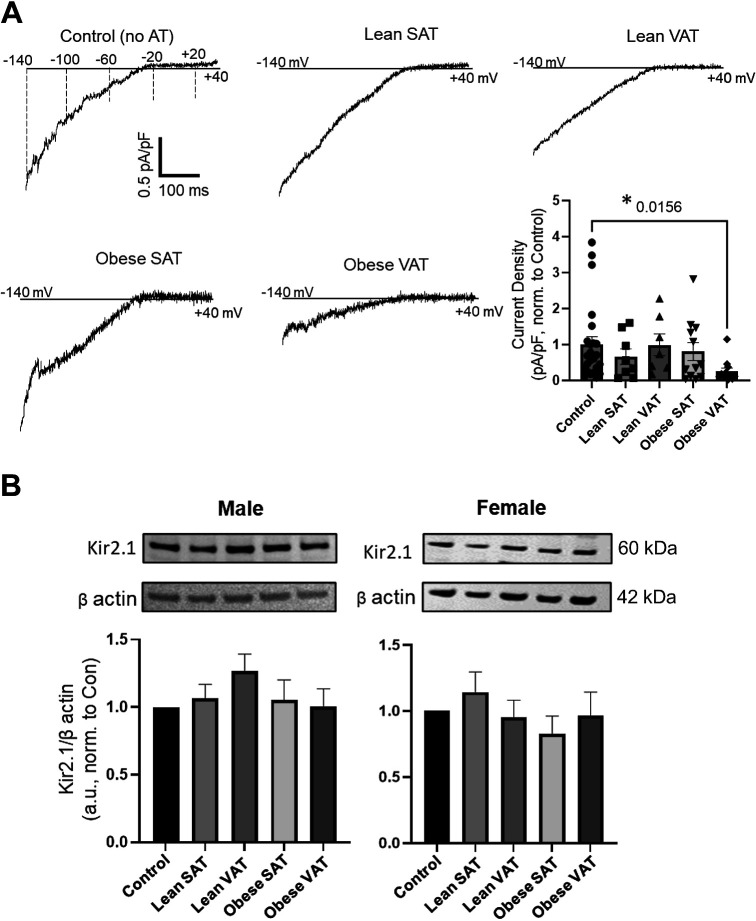
Visceral adipose tissue (VAT) from obese mice reduces endothelial Kir2.1 channel function in vitro. Human adipose microvascular endothelial cells (HAMECs) were incubated with AT from lean or obese mice for 48 h before assessing inwardly rectifying K^+^ currents via whole cell patch clamp. The holding potential was −30 mV, and the voltage ramp protocol was from −140 to +40 mV over 400 ms. *A*: representative current recordings from HAMECs from untreated control (*top left*) and cells exposed to subcutaneous adipose tissue (SAT) from lean mice (*top middle*), VAT from lean mice (*top right*), SAT from obese mice (*bottom left*), or VAT from obese mice (*bottom middle*). The representative control recording (*top left*) includes the voltage changes over time and reveals that the inwardly rectifying K^+^ currents reverse near the predicted reversal potential of approximately −21 mV under the specified patch conditions (i.e., 60 mM K^+^ bath, 140 mM K^+^ pipette solutions). Group data (*bottom right*) reveal normalized current densities (pA/pF) that were analyzed and compared at −100 mV to determine the effects of AT treatment on Kir2.1 function (*n* = 7–14 cells/AT group from 4 mice/diet group). *Significant differences relative to control as determined by the Kruskal-Wallis test followed by Dunn’s post hoc tests. AT treatment did not affect cell capacitance among groups (*P* = 0.6874). An equal number of male and female mice were used in these studies, and data were combined as no sex differences were detected when testing across sex within an AT group. *B*: representative Western blots and group data showing Kir2.1 expression in HAMECs following incubation with AT from either male or female mice (*n* = 8/group/sex). Untreated cells were used as controls. Kir2.1 expression densities were normalized to that of β-actin before analysis; a.u., arbitrary units.

To determine if VAT from obese mice induces endothelial Kir2.1 impairment, VAT and SAT were isolated from lean and obese mice following 8 wk of respective diets. HAMECs were then incubated with VAT or SAT (5 mg/mL) from either lean or obese mice for 48 h under standard culture conditions before assessing endothelial Kir2.1 currents via whole cell patch-clamp electrophysiology. Control cells did not receive adipose tissue (AT) treatment. Endothelial inwardly rectifying K^+^ currents, previously established to be largely conducted by Kir2.1 in human and murine adipose endothelial cells (11, 12), were recorded using a voltage ramp protocol of −140 to +40 mV over 400 ms in a 60 mM K^+^ bath. The representative control recording (Fig. 1*A*, *top left*) includes the voltage changes over time and reveals that the inwardly rectifying K^+^ currents reverse near the predicted reversal potential of approximately −20 mV under the specified patch conditions (i.e., 60 mM K^+^ bath and 135 mM K^+^ pipette solutions). Figure 1*A* also shows representative Kir current recordings from cells exposed to either SAT from lean mice (*top middle*), VAT from lean mice (*top right*), SAT from obese mice (*bottom left*), or VAT from obese mice (*bottom middle*) and reveals the *1*) similarities in Kir current densities between cells exposed to AT from lean mice and SAT from obese mice as compared to control, and *2*) reduction in Kir currents in cells exposed to VAT from obese mice as compared to control. Indeed, when normalized and compared to control Kir currents, only those cells exposed to VAT from obese mice exhibited significantly reduced Kir currents indicating that VAT impairs endothelial Kir2.1 in obesity (Fig. 1*A*, group data). These findings suggest that obesity-induced alterations specifically occurring in VAT may underlie endothelial Kir2.1 dysfunction in the local mesenteric arteries. For these studies, an equal number of male and female mice were used; however, no sex differences were detected, so the data were combined.

To determine if VAT from obese mice impairs Kir current densities by reducing channel expression, we tested the expression of Kir2.1 in HAMECs following exposure to AT from lean and obese mice via Western immunoblotting. Our past studies revealed that obesity does not influence endothelial Kir2.1 expression (11); therefore, we predicted that VAT from obese mice would not alter expression compared to the control. Indeed, there was no significant difference in Kir2.1 expression when comparing the AT-treated HAMECs to control, a finding that was observed in cells treated with AT from either male or female mice (Fig. 1*B*). These observations are in line with our previous studies and suggest that VAT may promote a functional impairment of Kir2.1 channels in obesity.

### VAT-Mediated Endothelial Kir2.1 Dysfunction Is Prevented by CD36 Downregulation

**Figure 2.**
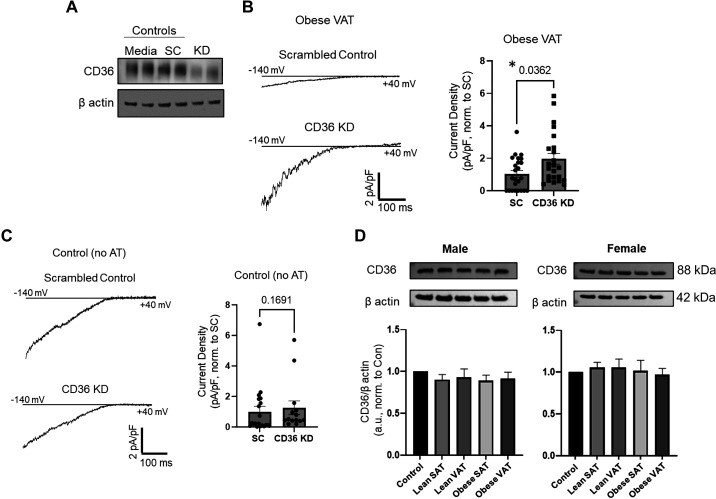
CD36 knockdown rescues endothelial Kir2.1 function in cells exposed to visceral adipose tissue (VAT) from obese mice. *A*: representative Western blot showing CD36 knockdown (KD) in human adipose microvascular endothelial cells (HAMECs) using siRNA as compared to control cells receiving the scramble siRNA (SC Control) or untreated cells (Media Control). Similar levels of CD36 KD were observed across 3 separate tests. *B*: CD36 was downregulated in HAMECs using siRNA before adipose tissue (AT) incubations and assessing inwardly rectifying K^+^ currents via whole cell patch clamp. Representative Kir current recordings from HAMECs exposed to VAT from obese mice and collected from cells receiving either the scrambled control (*top trace*) or CD36 siRNA (*bottom trace*). The holding potential was −30 mV, and the voltage ramp protocol was from −140 to +40 mV over 400 ms. Group data (*right*) reveal normalized current densities (pA/pF) that were analyzed and compared at −100 mV to determine the effects of CD36 KD on Kir2.1 function (*n* = 23–24 cells/group from 4 obese mice). *Significance as determined by a Mann-Whitney test. CD36 KD did not affect cell capacitance when compared to the scrambled control (SC; *P* = 0.4644). An equal number of male and female mice were used in these studies and data were combined as no sex differences were detected when comparing the effect of CD36 KD. *C*: representative Kir current recordings from HAMECs without AT and collected from cells receiving either the scrambled control (*top trace*) or CD36 siRNA (*bottom trace*). Group data (*right*) reveal normalized current densities (pA/pF) that were analyzed and compared at −100 mV to determine the effects of CD36 KD on Kir2.1 function (*n* = 15–20 cells/group). A Mann-Whitney test was used to test for significance. CD36 KD did not affect cell capacitance when compared to the scrambled control (*P* = 0.1966). Data were collected across 4 independent experiments. *D*: representative Western blots and group data showing CD36 expression in HAMECs following incubation with AT from either male or female mice (*n* = 8/group/sex). Untreated cells were used as controls. CD36 expression densities were normalized to that of β-actin before analysis using a Kruskal-Wallis test; a.u., arbitrary units.

Kir2.1 is inherently sensitive to the cellular lipid milieu and is inhibited by several lipid species including fatty acid derivatives (15). We therefore determined if VAT was mediating Kir2.1 dysfunction in a CD36-dependent fashion. As the major endothelial fatty acid translocase, endothelial CD36 was previously shown to be required for tissue utilization of fatty acids (24), yet its role in mediating endothelial dysfunction remains to be resolved. To determine if CD36 was required for VAT-induced Kir2.1 dysfunction, we downregulated CD36 expression in HAMECs using siRNA (Fig. 2*A*) before exposing HAMECs to VAT from obese mice and assessed endothelial Kir currents via whole cell patch-clamp electrophysiology. CD36 knockdown resulted in a significant recovery of Kir2.1 current density as compared to the scrambled control (Fig. 2*B*). In contrast, CD36 knockdown did not significantly affect Kir2.1 current density in cells that were not exposed to AT (Fig. 2*C*). Together, these findings suggest that endothelial CD36 is a mediator of VAT-induced Kir2.1 dysfunction in vitro.

We next tested the expression of CD36 in HAMECs following exposure to AT from lean and obese mice via Western immunoblotting to determine if VAT from obese mice increases CD36 expression as a potential contributor to Kir2.1 dysfunction. No differences in CD36 expression were detected in HAMECs exposed to AT isolated from male or female mice as compared to control (Fig. 2*D*). Taken together, these findings suggest that VAT may augment CD36 function and/or trafficking to the membrane to promote Kir2.1 impairment in obesity. Based on these findings, we next aimed to determine if VAT from obese mice induced an increase in CD36 function by assessing CD36-mediated fatty acid uptake.

### Endothelial Cells Exposed to VAT from Obese Mice Heavily Depend on CD36 for Fatty Acid Uptake

**Figure 3.**
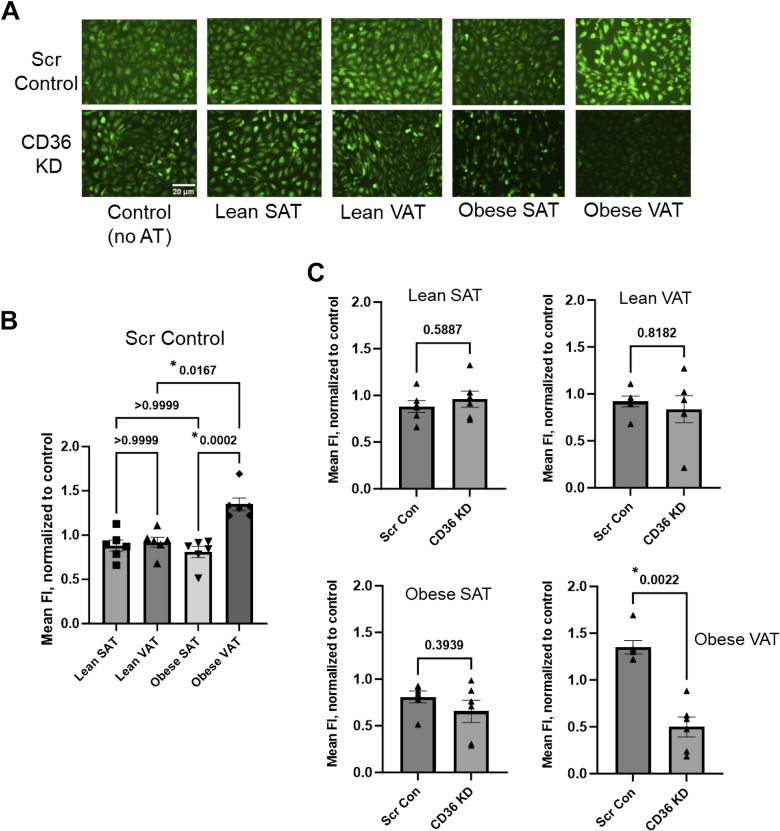
Human adipose microvascular endothelial cells (HAMECs) exposed to visceral adipose tissue (VAT) from obese mice heavily depend on CD36 for fatty acid uptake. *A*: representative fluorescent microscopy images (×20) showing internalized fluorescent fatty acid analog in HAMECs following adipose tissue (AT) treatments. Control cells were not incubated with AT. Before incubations with AT, cells received either the scrambled control (Scr) or CD36 siRNA to determine the effects of CD36 knockdown (KD) on fatty acid uptake. Scale bar = 20 μm. *B*: group data revealing the effects of AT on the mean fluorescence intensity (FI) in Scr-treated cells normalized to that of control cells that did not receive AT treatment (*n* = 6 independent experiments using 6 mice/group). *Significant differences between AT groups as determined by a Kruskal-Wallis test followed by Dunn’s post hoc tests. *C*: the effect of CD36 KD on fatty acid uptake was determined within each AT treatment group. The mean FI of cells that received either Scr or siRNA were normalized to the respective control group to assess the role of AT on CD36-mediated fatty acid uptake. *Significant differences in cells treated with subcutaneous adipose tissue (SAT) from lean mice (*top left*), VAT from lean mice (*top right*), SAT from obese mice (*bottom left*), and VAT from obese mice (*bottom right*) as determined by a Mann-Whitney test. No sex differences were detected when testing across sex within an AT group, so the data were combined.

To determine if VAT from obese mice induces an increase in endothelial CD36 function, HAMECs were incubated with a fluorescent fatty acid analog following exposure to AT from lean and obese mice. HAMECs were subsequently imaged via fluorescent microscopy to detect internalized fatty acids. Figure 3*A* shows representative images revealing fatty acid uptake in HAMECs treated with either the scrambled control or CD36 siRNA before AT treatments. Endothelial cells that received the scrambled control and were exposed to AT from lean or obese mice did not exhibit a significant difference in fatty acid uptake when compared to the control that did not receive AT treatment. Although not statistically significant, endothelial cells exposed to VAT from obese mice showed an average ∼35% increase in fatty acid uptake as compared to controls. However, as compared to cells exposed to VAT from lean mice and SAT from obese mice, cells exposed to VAT from obese mice exhibited a significantly greater level of fatty acid uptake (Fig. 3*B*), thereby suggesting a role specifically for VAT in inducing endothelial uptake of fatty acids in obesity.

Based on the evidence establishing endothelial CD36 as the major regulator of fatty acid uptake in vivo, we were surprised to find that CD36 knockdown did not significantly reduce fatty acid uptake in untreated control cells in vitro. The lack of an effect of CD36 knockdown was paralleled in cells exposed to AT from lean mice and SAT from obese mice where CD36 knockdown showed no effect on fatty acid uptake (Fig. 3*C*). In stark contrast, however, endothelial cells exposed to VAT from obese mice appeared to heavily rely on CD36 as CD36 knockdown resulted in a significant blunting (∼63%) of fatty acid uptake compared to the scrambled control-treated cells (Fig. 3*C*). These findings suggest that HAMECs in a culture likely employ alternative fatty acid uptake mechanisms to CD36, whereas VAT from obese mice promotes a transition to CD36-mediated fatty acid uptake, which is accompanied by enhanced uptake of fatty acids into endothelial cells in vitro. Together with the observation that CD36 knockdown rescues endothelial Kir2.1 function, an effect we previously showed is sufficient to restore endothelial function in obesity (11), our next goal was to determine the role of CD36 in obesity-induced endothelial dysfunction in arteries located in VAT using a well-established mouse model of diet-induced obesity.

### CD36 Ablation Prevents Obesity-Induced Endothelial Dysfunction in VAT Arteries and Restores the Kir/eNOS Axis

The loss of nitric oxide (NO) production in obesity-induced endothelial dysfunction is well-established; however, arteries located in distinct adipose depots are differentially affected. We and others have shown that VAT arteries exhibit robust endothelial dysfunction while SAT arteries retain endothelial function (5–8). We recently showed that obesity-induced VAT endothelial dysfunction is mediated by impairment of endothelial Kir2.1, critical upstream mediators of flow-induced NO production and vasodilation (5, 11). In contrast, SAT endothelial Kir2.1 remains functional in obesity and promotes NO-mediated vasodilation similarly to lean control counterparts. This dichotomy in endothelial and Kir2.1 (dys)function in obesity points to a VAT artery-specific pathophysiology that remains to be identified.

**Figure 4.**
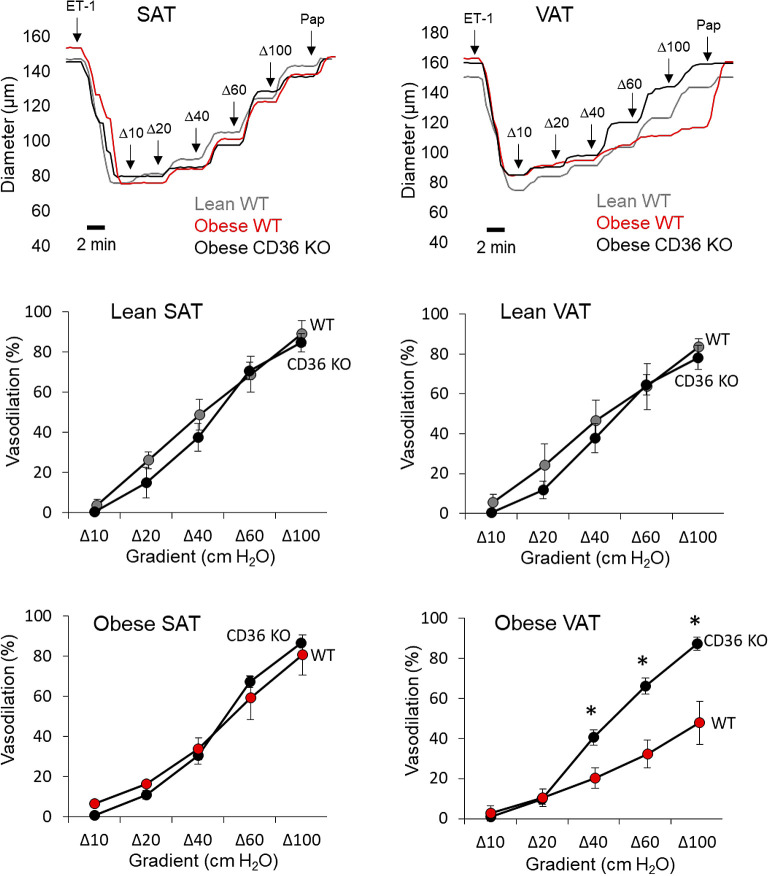
Flow-induced vasodilation is restored in visceral arteries of obese mice lacking CD36. Arteries from visceral adipose tissue (VAT) and subcutaneous adipose tissue (SAT) from wild-type (WT) and CD36 knockout (KO) mice were isolated and cannulated for pressure myography and the endothelial response to flow was measured as previously described (5, 22). Representative traces show the dilatory response to an increase in intraluminal flow in SAT and VAT arteries of lean and obese WT or obese CD36 KO mice. Preconstrictions to low doses of endothelin-1 (ET-1; ≤ 120 pM) were completed in under 2 min. Papaverine (Pap; 100 μM) was used to confirm the functionality of smooth muscle at the end of each protocol. Flow-induced vasodilation was assessed between WT and CD36 KO mice in arteries isolated from SAT of lean mice (*top left*), VAT of lean mice (*top right*), SAT of obese mice (*bottom left*), and VAT from obese mice (*bottom right*). *Significant differences as detected by a two-way ANOVA followed by Bonferroni post hoc tests (*n* = 5; 3 male and 2 female mice/group).

CD36 is a well-established mediator of atherogenesis and its role in numerous cell types, including vascular endothelium, has been identified (18, 25, 26). However, the role of CD36 in mediating the early stages of disease development, i.e., endothelial dysfunction, is unknown. Here we used a well-established mouse model of diet-induced obesity that we recently showed recapitulates the obese human condition in regard to VAT versus SAT endothelial function to determine the role of CD36 in mediating obesity-induced endothelial and Kir2.1 dysfunction in VAT arteries. WT and CD36 knockout (KO) mice were placed on a high-fat, Western diet (42% kcal from fat) for 8 wk, a time point in which robust VAT artery endothelial dysfunction is observed in obese mice (5, 11). Lean, age-matched controls were maintained on a standard rodent diet. To assess endothelial function, VAT and SAT arteries were isolated and subjected to pressure myography to determine the endothelial response to flow, an established physiological stimulus of Kir2.1 channels known to elicit NO production (12, 27, 28). Isolated arteries were cannulated and pressurized in specialized chambers before the arteries were preconstricted with endothelin-1. There were no significant differences in the baseline diameters of arteries between groups (Table 2). Step-wise increases in intraluminal flow were delivered using the pressure gradient method, as described previously (5, 22) Representative traces, generated by recording the arterial diameter every 30 s for the duration of the protocol, show that the dilatory response to an increase in intraluminal flow was rapid and reached a plateau within 2 min in SAT and VAT arteries from WT or CD36 KO mice (Fig. 4, *top*). As expected, arteries isolated from lean WT mice and SAT from obese WT mice responded to intraluminal flow by dilating on average between ∼77 and 84% of baseline at the maximum flow step whereas arteries isolated from VAT of obese WT mice exhibited blunted dilations to flow (∼45% of baseline at maximum flow; Fig. 4). Global CD36 ablation had no effect on the response to flow in arteries isolated from lean mice or in SAT from obese mice as compared to WT counterparts. However, arteries isolated from VAT of obese CD36 KO mice showed a complete and significant recovery of the dilatory response to flow (∼85% of baseline at maximum flow) as compared to VAT arteries from obese WT mice and similar to that observed in arteries of lean WT mice (Fig. 4), thereby supporting a role for CD36 in obesity-induced endothelial dysfunction in VAT arteries. These findings are further supported when observing the mean absolute diameter changes in response to an increase in flow (Table 3).

**Table 2.** Baseline AT artery diameters with and without inhibitors

Artery	Average Baseline Diameter ± SE, μm	Average Baseline Diameter following Incubation with Ba^2+^ ± SE, μm	Average Baseline Diameter following Incubation with l-NAME ± SE, μm
SAT-WT lean	123.3 ± 13.1	121.4 ± 14.2	122 ± 13.5
VAT-WT lean	120.8 ± 9.5	119.2 ± 10.0	118.6 ± 10.8
SAT-WT obese	115.3 ± 15.8	114.3 ± 16.1	112.8 ± 18.4
VAT-WT obese	117.7 ± 24.9	114.6 ± 22.8	115.0 ± 23.0
SAT-CD36 KO lean	125.5 ± 8.0	124.1 ± 7.3	122.9 ± 7.7
VAT-CD36 KO lean	138.6 ± 15.1	135.9 ± 16.1	135.8 ± 15.8
SAT-CD36 KO obese	145.1 ± 10.7	144.5 ± 10.7	144.4 ± 13.0
VAT-CD36 KO obese	134.1 ± 13.5	130.5 ± 12.5	129.8 ± 12.4

A two-way ANOVA was used when comparing baseline diameters across diet and genotype. One-way repeated measures ANOVAs were used to determine the effects of inhibitors within each artery group. AT, adipose tissue; SAT, subcutaneous adipose tissue; VAT, visceral adipose tissue; KO, knockout; WT, wild type; l-NAME nitro-l-arginine methyl ester.

**Table 3.** Mean arterial diameter increases in AT arteries in response to flow with or without inhibitors

Artery	Absolute Change in Artery Diameter at Δ100 Flow Gradient ± SE, μm	Absolute Change in Artery Diameter at Δ100 Flow Gradient with Ba^2+^ ± SE, μm	Absolute Change in Artery Diameter at Δ100 Flow Gradient with l-NAME ± SE, μm
SAT-WT lean	35.5 ± 9.8	16.5 ± 10.0† (*P =* 0.037)	14.1 ± 12.3† (*P =* 0.040)
VAT-WT lean	43.3 ± 6.4	25.6 ± 4.0† (*P =* 0.021)	23.9 ± 5.9† (*P =* 0.031)
SAT-WT obese	47.6 ± 5.3	31.8 ± 8.8† (*P =* 0.044)	33.6 ± 11.3† (*P =* 0.046)
VAT-WT obese	26.7 ± 4.3* (*P =* 0.025)	24.8 ± 4.6	27.0 ± 9.4
SAT-CD36 KO lean	47.0 ± 2.6	27.2 ± 4.7† (*P =* 0.002)	25.9 ± 4.4† (*P* < 0.001)
VAT-CD36 KO lean	45.5 ± 6.3	28.7 ± 3.4† (*P =* 0.008)	31.4 ± 5.3† (*P =* 0.009)
SAT-CD36 KO obese	45.5 ± 4.8	32.8 ± 4.5† (*P =* 0.006)	35.8 ± 4.3† (*P =* 0.011)
VAT-CD36 KO obese	54.8 ± 6.9# (*P =* 0.014)	29.6 ± 2.7† (*P =* 0.003)	32.5 ± 1.7† (*P =* 0.008)

Mean increases in diameters in response to flow were assessed relative to the preconstricted diameter in each artery group. A two-way ANOVA followed by Bonferroni post hoc testes was used when comparing baseline diameters across diet and genotype (*significantly different vs. VAT-WT lean; #significantly different vs. VAT-WT obese). One-way repeated measures ANOVAs followed by bonferroni post hoc tests were used to determine the effects of inhibitors within each artery group (†significantly different vs. diameter changes in the absence of inhibitor within an artery group). AT, adipose tissue; SAT, subcutaneous adipose tissue; VAT, visceral adipose tissue; KO, knockout; WT; wild type; l-NAME, nitro-l-arginine methyl ester.

**Figure 5.**
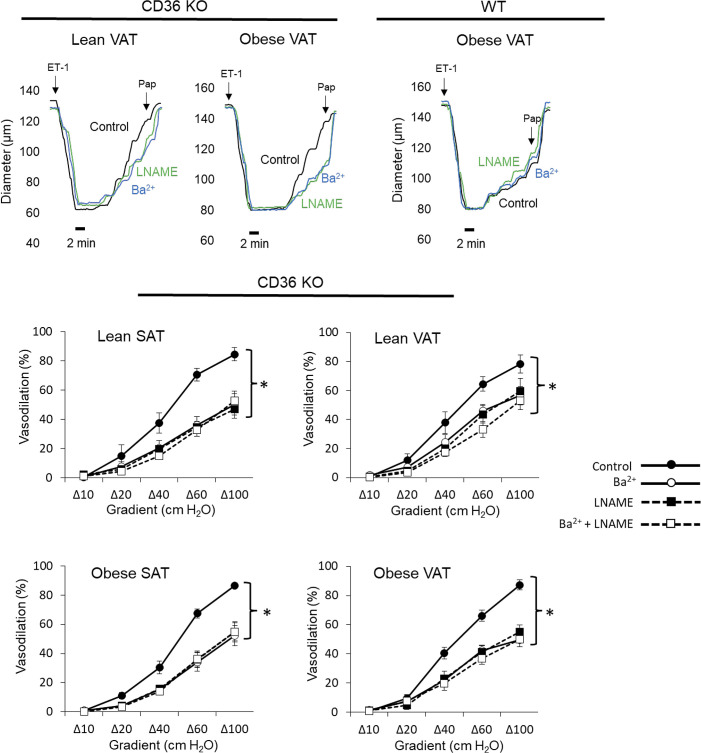
CD36 ablation restores flow-induced vasodilation in visceral arteries of obese mice via recovery of Kir2.1/endothelial nitric oxide synthase (eNOS) function. Representative traces show the dilatory response to an increase in intraluminal flow in visceral adipose tissue (VAT) arteries of lean and obese CD36 knockout (KO) mice. A representative trace generated from an obese wild-type (WT) mouse VAT artery is also shown for comparison. Preconstrictions to low doses of endothelin-1 (ET-1; ≤120 pM) were complete in under 2 min. Papaverine (Pap; 100 μM) was used to confirm the functionality of smooth muscle at the end of each protocol. The effects of Kir and eNOS inhibition on flow-induced vasodilation were measured in arteries of CD36 KO mice isolated from subcutaneous adipose tissue (SAT) of lean mice (*top left*), VAT of lean mice (*top right*), SAT of obese mice (*bottom left*), and VAT from obese mice (*bottom right*). Following initial responses to intraluminal flow, VAT and SAT arteries from CD36 KO mice were sequentially incubated with BaCl_2_ (30 μM; Kir channel inhibitor), nitro-l-arginine methyl ester (l-NAME; 100 μM; eNOS inhibitor), and then a combination of both inhibitors for 30 min before reassessing dilations to flow. The inhibitors were washed out of the circulating bath chamber between each assessment of the dilatory response to flow. *Significant differences between the initial response to flow and pharmacological treatments as detected by repeated measures two-way ANOVA followed by Bonferroni post hoc tests (*n* = 5; 3 male and 2 female mice/group).

To determine if CD36 ablation rescues VAT artery endothelial function by restoring the Kir2.1/eNOS axis, we next determined if blocking Kir channels with Ba^2+^ (30 μM) or eNOS with l-NAME (100 μM) inhibited dilations to flow in arteries isolated from AT of CD36 KO mice. Following the initial assessment of the dilatory response to flow, AT arteries from lean and obese CD36 KO mice were then incubated with either inhibitor alone or in combination for 30 min before reassessing endothelial function, an approach we previously established results in blunted dilations to flow in AT arteries from lean WT mice and in SAT arteries from obese WT mice. In contrast and highlighted in representative traces shown in Fig. 5, the remaining dilatory response to flow in VAT arteries from WT obese mice is unaffected by Kir2.1 or eNOS inhibition, further indicating impairment of this signaling pathway (5, 11). Both Kir2.1 and eNOS inhibition alone and in combination resulted in significantly reduced dilations to flow in each AT artery type from CD36 KO mice (Fig. 5 and Table 3) indicating that CD36 ablation *1*) had no effect on endothelial or Kir2.1 function in arteries from lean mice AT or SAT arteries of obese mice as the pretreatment response to flow was comparable to WT controls (Fig. 4) and is similarly inhibited by Ba^2+^ and l-NAME, and *2*) restored the Kir2.1/eNOS axis in otherwise dysfunctional VAT arteries of obese mice. These findings suggest a role for CD36 in mediating obesity-induced endothelial dysfunction in VAT arteries by disrupting Kir2.1 and eNOS signaling. In addition, there were no effects of Ba^2+^ or l-NAME on baseline arterial diameters within artery groups (Table 2).

## DISCUSSION

Adipose tissue is now recognized as more than the major site of lipid storage, and its role as an endocrine and paracrine tissue has garnered much interest in recent years. The contributions of AT in governing vascular tone under pathological conditions have also been a major focal point, as obesity-induced endothelial dysfunction represents a major precursor to the development of severe cardiovascular disease. Interestingly, VAT depots have been shown to exhibit an elevated inflammatory state as compared to SAT in obese individuals, suggesting that specific alterations in VAT occur under obesogenic conditions (2–4). This dichotomy is paralleled by observations revealing that VAT arteries exhibit endothelial dysfunction whereas arteries embedded in SAT retain endothelial function, suggesting that alterations to VAT in obesity may have a deleterious impact on the local vasculature (5–8); however, this has yet to be fully elucidated.

In the present study, we used an in vitro model whereby human adipose microvascular endothelial cells in culture were incubated with AT from lean or obese mice to determine the effects of AT on endothelial Kir2.1 channel function, a critical component of endothelium-dependent NO production and vasodilation well-known to be disrupted in obesity (17, 28), which we recently showed to be impaired in endothelium of VAT arteries isolated from obese mice and humans (5, 11). Our findings indicate that VAT from obese mice robustly decreases endothelial Kir2.1 currents, effects that do not seem to be mediated by a decrease in channel expression. As obesity is strongly associated with an increase in circulating free fatty acids (17), cellular derivatives of which are established inhibitors of Kir2.1(15, 16), we further hypothesized that VAT-mediated impairment of Kir2.1 is dependent on an increase in endothelial CD36 function. We found that CD36 downregulation rescued endothelial Kir2.1 currents in cells exposed to VAT from obese mice in vitro. In contrast, we did not observe an effect of CD36 knockdown on Kir2.1 currents in cells that were not exposed to AT, suggesting a VAT-induced upregulation of CD36 that impairs Kir2.1 in obesity. We further revealed that endothelial cells exposed to VAT in obese mice had a marked increase in CD36-dependent fatty acid uptake as compared to cells exposed to AT from lean mice and SAT from obese mice further supporting a VAT-mediated increase in CD36 function that may underlie the impairment of endothelial Kir2.1. The importance of CD36 in mediating obesity-induced endothelial dysfunction in VAT arteries was additionally supported by our ex vivo studies where CD36 ablation restored VAT artery endothelial function in a Kir2.1/eNOS-dependent manner. Together, our findings reveal a new role for CD36 in obesity-induced endothelial dysfunction that is likely driven by VAT and results in Kir2.1 and endothelial dysfunction.

### The Role of Endothelial Kir2.1 in Flow-Induced Vasodilation

Several recent studies have demonstrated that endothelial Kir2.1 is an essential contributor to endothelium-dependent vasodilation in response to both chemical and mechanical cues likely through distinct signaling mechanisms (11, 12, 29–31). For instance, Sonkusare et al. (29) recently revealed that Kir2.1 channels act as “boosters” of the dilatory response when, upon stimulation of receptor-mediated pathways that result in the activation of Ca^2+^-activated K^+^ channels (K_Ca_), the ensuing hyperpolarizing potentials promote the activation of Kir2.1, further eliciting release of K^+^ from the endothelium and effectively contributing to endothelium-derived hyperpolarization (EDH). However, in response to fluid shear, we recently showed that Kir2.1 activation and contributions to endothelium-dependent vasodilation are dependent on the glycocalyx upstream of Akt/eNOS/NO signaling [i.e., endothelium-derived relaxing factor (EDRF) signaling as opposed to EDH (5, 11, 12)], although how the glycocalyx regulates Kir2.1 activity [e.g., through possible tethering interactions (32)] and how activation of Kir2.1 results in Akt activation to ultimately promote an increase in eNOS activity remain unclear.

In contrast to the receptor-mediated mechanism where Kir2.1 and K_Ca_ are proposed to work in concert, it appears that in response to a mechanical stimulus such as shear stress, these pathways are operated in parallel. This is evidenced in our previous studies where *1*) inhibition of Kir2.1 with Ba^2+^ does not influence the contributions of K_Ca_ to vasodilation, *2*) blocking small conductance K_Ca_ with apamin does not appear to impede Kir2.1 contributions to vasodilation, yet *3*) a combination of Kir2.1 and K_Ca_ blockade drastically reduces dilations to flow (12, 23). It is certainly reasonable to suspect that flow-activation of Kir2.1 would also contribute to EDH and/or electrical coupling to smooth muscle independent of NO as these channels are established contributors to the conducted vasomotor response via endothelial-smooth muscle gap junctions (33) and were recently confirmed to be expressed at the myoendothelial junction (34). However, in our studies the inhibition of Kir2.1 and eNOS, separately and in combination, elicited similar deficits to the dilatory response to flow in AT arteries (11, 12, 23) perhaps suggesting that *1*) an extremely low number of channels in AT endothelium (12, 29) do not sufficiently hyperpolarize the endothelium to, in turn, hyperpolarize and relax smooth muscle, and/or *2*) distinct populations of Kir2.1 contribute to unique signaling pathways promoting various forms of endothelium-dependent vasodilation.

While mechanisms governing the contributions of endothelial Kir2.1 channels to either EDH or EDRF under distinct stimuli remain unclear, it may be driven by apical (driving EDRF with the glycocalyx in the lumen independent of K_Ca_) versus basal (driving EDH with K_Ca_ at the basolateral membrane) surface expression of the channel. Although not supported by our studies per se, whether or not a population of Kir2.1 channels is activated following flow-activation of K_Ca_ similarly to the receptor-mediated pathway that would contribute to flow-mediated EDH remains to be fully elucidated. Future studies should be aimed a delineating surface expression of Kir2.1 in the context of these possible roles. As we recently showed that obesity does not appear to influence the contributions of K_Ca_ channels to the dilatory response that remained in VAT arteries, which was unaffected by Kir2.1 inhibition and could be restored with endothelial overexpression of Kir2.1 (5, 11), we focused on Kir2.1 in the present study.

### VAT as an Upstream Mediator Driving Kir2.1 and Endothelial Dysfunction in Obesity

Previous studies identified that parenchymal AT influences K^+^ ion channels to regulate vascular tone of local arteries. Perivascular AT (PVAT) from lean or obese swine was shown to differentially inhibit distinct coronary artery K^+^ channels (35). Specifically, PVAT from lean swine inhibited K_Ca_ and K_v_ channels while PVAT from obese swine had no effect on these K^+^ channels but instead inhibited K_ATP_ channels, members of the Kir family, in coronary arteries from both lean and obese swine. The authors did not detect significant changes in coronary artery expression with these channels between lean and obese swine, suggesting that there is likely a switch in the production of adipose metabolites and/or adipokines with obesity that results in a paracrine-mediated functional downregulation of different K^+^ channels. Similarly, we show that VAT from obese mice has the capacity to reduce Kir2.1 currents; however, we did not observe this effect in cells treated with AT from lean mice or SAT from obese mice, providing additional evidence that distinct K^+^ channels are uniquely and dynamically influenced by AT. We also found that AT does not affect endothelial Kir2.1 expression, which further supports a modification of channel activity and/or trafficking to the membrane suggesting that VAT is altered by obesity and may influence the local endothelium. Collectively, these findings reveal the regulation of vascular K^+^ ion channels by specific AT depots is altered under obesogenic conditions.

Numerous studies have investigated the effects of PVAT on vascular function in obesity with several groups identifying PVAT-derived reactive oxygen species (ROS) as a driving force in endothelial dysfunction (36). While we did not directly test the role of AT-derived ROS in the present study, a variety of mechanisms including increased fatty acid accumulation in the endothelium are established to promote elevated ROS production (17), which may underlie CD36-mediated impairment of Kir2.1 in several ways. First, we recently showed that endothelial Kir2.1 is regulated by the glycocalyx, which is physically disrupted in mesenteric arteries isolated from VAT of obese mice (11). Similar findings were observed in the aortas of obese mice pertaining to disruption of the glycocalyx (37), and other studies have shown that aortic PVAT induces endothelial dysfunction (38), thereby offering a potential link between PVAT, disruption of the glycocalyx, and impairment of K^+^ channels under obesogenic conditions. The glycocalyx is inherently sensitive to oxidative stress, which promotes its degradation (39, 40) and may therefore underlie Kir2.1 dysfunction in obesity. Therefore, an increase in endothelial fatty acid uptake/accumulation that would promote elevated ROS production may indirectly induce Kir2.1 dysfunction by degrading the glycocalyx. Kir2.1 may also be directly inhibited by ROS as it possesses extracellular cysteines similar to other Kir channel family members shown to be inhibited by oxidizing agents through modification of these residues (41, 42). Therefore, future studies investigating the potential role and source (i.e., VAT vs. endothelium) of ROS in VAT-mediated impairment of Kir2.1 are warranted. However, our present in vitro findings support a role for endothelial CD36 as a mediator of Kir2.1 dysfunction that would require such effects to occur at the endothelium.

Elevated circulating free fatty acids in obesity may also result in the impairment of Kir2.1 independent of ROS. Another mode of possible VAT-mediated inhibition of Kir2.1 that may be facilitated by CD36 is the metabolic conversion of intracellular fatty acids into metabolite derivatives including long-chain fatty acyl CoA-esters and ceramides that may inhibit the channel directly or indirectly through cell signaling events, respectively (15, 16). An increase in fatty acid accumulation may result in an increase in one or more of these lipid products that would promote Kir2.1 inhibition. The present study supports this as a possible mechanism as VAT from obese mice appeared to invoke a transition in endothelial cells to heavily rely on CD36-mediated fatty acid uptake, which was elevated as compared to cells exposed to other AT groups in vitro. However, CD36 is a known scavenger receptor with many ligands including oxidized low-density lipoprotein, apoptotic cells, and thrombospondin that can trigger a variety of downstream signaling cascades (18, 26). This alludes to a potential complex role for VAT-induced upregulation of CD36 in mediating Kir2.1 impairment in obesity in vivo although our studies in isolated arteries of CD36 KO mice further support such a role is present. Future studies will be aimed at deciphering the exact mechanisms of *1*) VAT-mediated upregulation of CD36, and *2*) CD36-mediated impairment of endothelial Kir2.1 in obesity.

### CD36 and Obesity-Induced Endothelial Dysfunction in VAT Arteries

CD36 is a well-established contributor to the development of atherosclerosis yet its role in mediating early stages of disease processes remains unknown. Here we determined that global CD36 ablation rescues VAT artery endothelial function in obese mice via restoring Kir2.1/eNOS signaling in the endothelial response to flow. Loss of Kir2.1 function results in blunted NO production and endothelial dysfunction, and we recently showed that overexpression of Kir2.1 specifically in VAT vascular endothelium was able to restore endothelial function in mesenteric arteries of obese mice via restoring eNOS-mediated NO production (11). As we observed a similar recovery of Kir2.1 and eNOS function in VAT arteries of obese mice lacking CD36, these findings suggest that CD36 may be a useful target in preventing the progress to more severe disease states by restoring endothelial function in afflicted vasculature.

Findings from our in vitro studies indicate that endothelial CD36 may be the major mediator of Kir2.1 impairment in the presence of VAT. However, the use of a global CD36 KO mouse model does not allow us to differentiate between specific cell types by which the absence of CD36 may be inducing the recovery in VAT artery endothelial function in vivo. In addition to vascular endothelium, CD36 is expressed in adipocytes and immune cells located in AT, each of which may play a role in inducing endothelial dysfunction in obesity (43–45). In addition, these studies in particular do not necessarily detail a role for VAT versus SAT in obesity-induced endothelial dysfunction; however, they further support a role for CD36 as a major player upstream of Kir2.1 impairment. Based on our present findings, future studies will focus on the role of endothelial CD36-mediated endothelial dysfunction in obesity and decipher the role of VAT and SAT in vivo.

### Conclusions

Our findings show that spatially distinct adipose depots, specifically the abdominal SAT and mesenteric VAT, differentially affect endothelial Kir2.1 in vitro in obesity and provide new insight into the potential role of CD36 in mediating Kir2.1 and endothelial dysfunction. As such, CD36 may serve as a target in restoring endothelial function to prevent the progress of cardiovascular disease by rescuing Kir2.1-mediated NO production. Future studies should be aimed at determining the specific mechanisms by which VAT regulates CD36 in endothelium and potentially other AT cells as well as how CD36 promotes Kir2.1 impairment in obesity-induced endothelial dysfunction.

## DATA AVAILABILITY

Data will be made available upon reasonable request.

## GRANTS

The research reported in this publication was supported by an Institutional Development Award (IDeA) from National Institute of General Medical Sciences Grant 2P20GM113125.

## DISCLOSURES

No conflicts of interest, financial or otherwise, are declared by the authors.

## AUTHOR CONTRIBUTIONS

S.R. and I.S.F. conceived and designed research; S.R., M.S., E.C.H., E.J.J., T.N., and I.S.F. performed experiments; S.R., M.S., E.C.H., E.J.J., T.N., and I.S.F. analyzed data; S.R. and I.S.F. interpreted results of experiments; S.R., M.S., E.C.H., E.J.J., T.N., and I.S.F. prepared figures; S.R., M.S., E.C.H., E.J.J., T.N., and I.S.F. drafted manuscript; S.R., M.S., E.C.H., E.J.J., T.N., and I.S.F. edited and revised manuscript; S.R., M.S., E.C.H., E.J.J., T.N., and I.S.F. approved final version of manuscript.
